# SMOKING IMPACTS *HELICOBACTER PYLORI* ERADICATION: RESULTS FROM A BRAZILIAN UNIVERSITY HOSPITAL

**DOI:** 10.1590/S0004-2803.24612025-059

**Published:** 2025-10-27

**Authors:** Amanda Avesani Cavotto Furlan Relva da FONTE, Marlone CUNHA-SILVA, Michelle Viviane Sá dos Santos RONDON, Cristina Rodrigues dos SANTOS, Natalicia Hifumi HARA, Cristiane Kibune NAGASAKO

**Affiliations:** 1 Universidade Estadual de Campinas, Faculdade de Ciências Médicas, Departamento de Clínica Médica, Campinas, São Paulo, Brasil.; 2 Universidade Estadual de Campinas, Laboratório de Hepatologia e Gastroenterologia, Gastrocentro, Campinas, São Paulo , Brasil.

**Keywords:** Helicobacter pylori, therapeutic failure, smoking, Teste respiratório, erradicação *Helicobacter pylori*, tabagismo

## Abstract

**Introduction:**

*Helicobacter pylori* infection has a high prevalence in Brazil, and eradication therapy is recommended for all diagnosed cases. While smoking has been identified as a factor associated with therapeutic failure, its impact on treatment efficacy has not yet been conclusively demonstrated in studies conducted within the Brazilian population.

**Objective:**

To determine the factors associated with *Helicobacter pylori* eradication failure in patients submitted to empirical 14-day antimicrobial therapy with omeprazole 20 mg BID, amoxicillin 1 g BID, and clarithromycin 500 mg BID at a Brazilian university center.

**Methods:**

An observational and retrospective study was conducted in the Hospital de Clinicas, University of Campinas, Brazil, between April 2022 and September 2023. The study included treatment-naive patients with *Helicobacter pylori* infection. These patients received PPI-clarithromycin triple therapy and underwent the 13C-urea breath test (13C-UBT) to evaluate eradication. Clinical and demographic variables were analyzed as potential factors associated with therapeutic failure, including age, gender, obesity, diabetes mellitus, anxiety and depression disorders, irritable bowel syndrome, fibromyalgia, smoking, alcoholism and cirrhosis.

**Results:**

A total of 97 patients were included, with a mean age of 55±14 years, 67 (69.1%) female. The antimicrobial treatment achieved 90.7% effectiveness (n=88). Smoking (*P*=0.0068) was the only factor associated with eradication failure after multiple regression analysis.

**Conclusion:**

Smoking was the only factor associated with *Helicobacter pylori* eradication failure, as determined by the 13C-urea breath test.

## HIGHLIGHTS

• The present study aims to identify factors associated with *Helicobacter pylori* eradication failure within a local population cohort.

• This is a retrospective study in a Brazilian university hospital assessing factors influencing eradication failure in treatment-naive patients using PPI-clarithromycin triple therapy.

• The study included 97 patients, with smoking identified as the sole factor significantly linked to therapeutic failure.

• The study concludes that smoking is the only independent predictor of *Helicobacter pylori* eradication failure in this Brazilian cohort.

## RESUMO

**Contexto:**

A infecção por *Helicobacter pylori* apresenta alta prevalência no Brasil, sendo a terapia de erradicação indicada para todos os casos diagnosticados. Embora o tabagismo esteja relacionado à falha terapêutica, a influência desse fator na eficácia do tratamento ainda não foi demonstrada em estudos conduzidos na população brasileira.

**Objetivo:**

Determinar os fatores associados à falha na erradicação da *Helicobacter pylori* em pacientes submetidos à terapia antimicrobiana empírica de 14 dias com: omeprazol 20 mg duas vezes ao dia, amoxicilina 1 g duas vezes ao dia e claritromicina 500 mg duas vezes ao dia ao dia, em um centro universitário no Brasil.

**Métodos:**

Trata-se de estudo observacional e retrospectivo que incluiu pacientes atendidos no Ambulatório de Gastroenterologia do Hospital de Clínicas da Universidade Estadual de Campinas, Brasil, virgens de tratamento, que receberam tratamento antimicrobiano para *Helicobacter pylori* (terapia tripla com claritromicina) e foram submetidos ao teste respiratório com 13C-ureia para controle da erradicação, entre abril de 2022 e setembro de 2023. Foram analisadas variáveis clínicas e demográficas como potenciais fatores associados à falha terapêutica, incluindo idade, sexo, obesidade, diabetes mellitus, transtornos de ansiedade e depressão, síndrome do intestino irritável, fibromialgia, tabagismo, etilismo e cirrose.

**Resultados:**

Foram incluídos 97 pacientes, com média de idade de 55±14 anos, 67 (69,1%) do sexo feminino. O tratamento antimicrobiano foi eficaz em 90,7% (n=88). Tabagismo (*P*=0,0068) foi o único fator associado à falha de erradicação após análise multivariada.

**Conclusão:**

Tabagismo foi o único fator associado à falha na erradicação da *Helicobacter pylori*, identificado pelo teste respiratório com 13C-ureia como método diagnóstico.

## INTRODUCTION

*Helicobacter pylori* infection is highly frequent in Brazil, especially in the Northern and Northeastern regions, with prevalence rates reported of up to 75%^1^.

Chronic *H. pylori* infection induces persistent inflammation of the gastric mucosa, leading to gastritis, peptic ulcers, mucosal atrophy, and gastric malignancies. Additionally, it has been implicated in the pathophysiology of several extra-gastrointestinal disorders, including immune thrombocytopenic purpura and chronic spontaneous urticaria^2^.

*H. pylori* eradication (HpE) therapy is recommended to all diagnosed cases. Because limited and contrasting data from developing countries are available (one Brazilian report to date)^3^, the efficacy of treatment regimens remains poorly understood. The present study aims to identify factors associated with HpE failure within a local population cohort.

## METHODS

### Study design

This is an observational, retrospective and single-center study, including treatment-naive patients submitted to the 13C-UBT to confirm *H. pylori* post-treatment eradication, assisted in the Hospital de Clinicas, University of Campinas, Southeastern Brazil, between April 2022 and September 2023. The study included patients aged 18 to 84 years with a previous *Helicobacter pylori* diagnosis confirmed by histopathological testing and no prior treatment. The only exclusion criteria was phenylketonuria. No patients were excluded. Complete adherence to the prescribed antimicrobial regimen was documented in all patients.

Clinical-epidemiological features of the patients were described: age, gender, body mass index and comorbidities (obesity, diabetes mellitus, anxiety and depression disorder, irritable bowel syndrome, fibromyalgia, alcohol use disorder, smoking and cirrhosis).

### Study population

#### Variables were defined as following:

Obesity: body mass index ≥ 30 kg/m².

Diabetes mellitus: current use of antidiabetic medication or HbA1C>5.7%.

Anxiety and depression disorder: current use of antidepressant or anxiolytic medication.

Irritable bowel syndrome: presence of abdominal pain at least one day per week in the last three months, associated with two or more of the following: related to defecation, associated with a change in stool frequency, or asso­ciated with a change in stool form - according to Rome IV criteria.

Fibromyalgia: widespread pain lasting for at least three months and the presence of other symptoms like fatigue and cognitive difficulties, wi­thout any other underlying condition that could ex­plain the symptoms - according to the American Colle­ge of Rheumatology criteria.

Alcohol use disorder: habitually drinking more than one alcoholic drink per day.

Smoking: habitually smoking more than one cigarette per day. 

Cirrhosis: confirmed by imaging or biopsy findings, according to medical records.

According to the IV^TH^ Brazilian Consensus Conference on *Helicobacter pylori* infection^4^, in the absence of susceptibility testing, a 14-day triple therapy regimen combining proton pump inhibitors, amoxicillin, and clarithromycin is recommended as the first-line treatment. In the present study, the following empirical eradication regimen for *H. pylori* was prescribed in all cases:

 • 14 day omeprazole 20 mg BID + amoxicillin 1 g BID + clarithromycin 500 mg BID.

### Urea breath test

The breath samples were collected using the IDkit HPTM Two Test and analyzed by the Exalenz BreathID® Hp *H. pylori* Breath Test System. All patients were advised to stop taking antibiotics and PPIs for at least four weeks before the test.

Samples were collected at baseline and 15 minutes after ingestion of 75 mg of 13C-urea, reconstituted with 4.3 g of powdered Citrica (citric acid) and 150 mL of filtered water. Subjects fasted for one hour before the test.

The breath samples were analyzed using the BreathID® Hp Lab equipment software and molecular correlation spectrometry (MCS™). A delta over baseline (DOB) more significant than five was considered positive^5^.

### Statistical analysis

Quantitative variables were described in terms of their means and standard deviations, while categorical variables were described in terms of their proportions. Associations were assessed using the chi-square or Fisher’s exact test for categorical variables and Mann-Whitney for numerical variables. Simple and multiple logistic models applying stepwise regression analyzed the factors associated with HpE.

*P*-values < 0.05 were considered statistically significant. The statistical package SAS System version 9.4 for Windows software was used for statistical analysis.

## RESULTS

A total of 97 patients were included (Table 1). The mean age was 55±14 years; 67 (69.1%) were female. Twenty-eight had obesity (28.8%); 27.8% (n=27) diabetes; 26.8% (n=26) anxiety and depression disorder; 14.4% (n=14) cirrhosis and 12.3% (n=12) were current smokers.

**TABLE 1:** Clinical-epidemiological profile of the sample and comparison between *H. pylori* negative and *H. pylori* positive groups.

Clinical Features	n(%) or mean (±SD)	***H. pylori* negative (n=88)**	***H. pylori* positive (n=9)**
		n (%) or mean (±SD)	n (%) or mean (±SD)
Age (year)	55 ±14 **n=97**	55 (±14)	51 (±13)
Female (sex)	67 (69.1%)	59 (67%)	8 (88.9%)
BMI (Kg/m²)	27.5 (±5.3)	27.6 (±5.5)	26.1 (±3.5)
Obesity (BMI≥30kg/m²)	28 (28.8%)	27 (30.7%)	1 (11.1%)
Diabetes mellitus	27 (27.8%)	26 (29.5%)	1 (11.1%)
Anxiety and depression disorder	26 (26.8%)	22 (25%)	4 (44.4%)
Irritable bowel Syndrome	4 (4.1%)	4 (4.50%)	0 (0%)
Fibromyalgia	4 (4.1%)	2 (2.3%)	2 (22.2%)
Alcohol use disorder	3 (3%)	3 (3.4%)	0 (0%)
Smoking	12 (12.3%)	8 (9.1%)	4 (44.4%)
Cirrhosis	14 (14.4%)	11 (12.5%)	3 (33.3%)

BMI: body mass index; CI: confidence interval; OR: odds ratio; SD: standard deviation. ¹ based on Mann-Whitney test /² based on Chi-square test /³ based on Fisher’s exact test.

The antimicrobial treatment achieved 90.7% effectiveness (n=88). Fibromyalgia (*P*=0.0196) and smoking (*P*=0.0067) were associated with eradication failure after simple regression analysis. However, in the multiple regression analysis, only smoking (*P*=0.0068) remained significant (Table 2).

**TABLE 2:** Results of simple and multiple logistic regression analysis assessing factors associated with *H. pylori* eradication.

Variable	Categories	***P*-value**	OR	CI95%
SIMPLE REGRESSION				
Sex	Female x Male	0.2068	0.254	0.030-2.131
Obesity (BMI≥30kg/m²)	No x Yes	0.2441	0.282	0.034-2.371
Diabetes mellitus	No x Yes	0.2651	0.298	0.035-2.505
Anxiety and/or depression disorder	No x Yes	0.2205	2.400	0.592-9.738
Fibromyalgia	No x Yes	***0.0196***	**12.286**	**1.496-100.923**
Smoking	No x Yes	***0.0067***	**8.000**	**1.781-35.935**
Cirrhosis	No x Yes	0.1069	3.500	0.763-16.053
MULTIPLE REGRESSION				
Smoking	No x Yes	**0.0068**	**13.016**	**2.027-83.598**

BMI: body mass index; CI: confidence interval; OR: odds ratio.

## DISCUSSION

The urea breath test with carbon isotope 13 (13C-UBT) is the gold-standard noninvasive diagnostic method for *H. pylori* infection, especially in eradication control^6^. The test has been validated in Brazil in adults and children aged six and over, with sensitivity and specificity above 95%^7^. The high sensitivity is due to its ability to assess the stomach as a whole, unlike methods based on sample analysis, since *H. pylori* gastric distribution can be heterogeneous. False-negative results can be found in special situations, such as a low bacterial concentration in the gastric lumen, rapid gastric emptying, previous gastric surgery, and recent administration of antibiotics, bismuth and proton pump inhibitors. False positive results have rarely been described in the presence of other urease-producing microorganisms in the gastric lumen, or even in the oral cavity^8^. However, the statistical relevance of these findings is considered to be very limited.

Patient preferences play a critical role in the selection of diagnostic tests for *H. pylori* infection. Compared to invasive procedures, non-invasive tests such as the 13C-UBT are generally well-tolerated, causing minimal discomfort, which increases patient satisfaction. The 13C-UBT is safe and has a higher cost-benefit ratio over upper digestive endoscopy^9^.

The failure rate of HpE is increasing. The most common causes are antibiotic resistance and poor adherence to medical therapy. However, the impact of smoking-a host-related factor-on HpE remains unclear. While some studies suggest that smoking increases treatment failure rates^10^ ^,^ ^11^, others have found no significant association^12^.

The only other Brazilian study that evaluated potential factors associated with *Helicobacter pylori* treatment failure^3^ (n=97), found no correlation with age, smoking, or alcohol consumption. However, it identified a positive association with clarithromycin resistance, cagA-negative status, and male gender.

In this study, smoking was the only factor associated with eradication failure. Chronic exposure to cigarette smoke components triggers several pathophysiological mechanisms, including increased secretion of hydrochloric acid and pepsin, impaired blood flow to the gastric mucosa, reduced bioavailability and efficacy of antibiotics, and enhanced CYP2C19 enzyme activity, which accelerates the metabolism of PPIs^13^. Additionally, smoking is associated with poor adherence to treatment regimens, increased antibiotic resistance, a higher bacterial load, and a propensity for infection with high-virulence *H. pylori* strains^14^.

This is the first Brazilian study to identify smoking as a significant factor associated with HpE failure, highlighting the importance of addressing tobacco use in the management of *H. pylori* infection to improve treatment outcomes in our population.

### Limitations

This retrospective study did not include HpE comparison with histopathological examination. However, the 13C-urea breath test was considered a reference standard, given its high sensitivity and specificity. 

All patients received empirical treatment without prior resistance testing. Therefore, the presence of antibiotic-resistant strains among those who failed treatment cannot be excluded; in such cases, treatment failure may be attributed to antibiotic resistance rather than other contributing factors.

## CONCLUSION

Smoking was the only factor associated with *Helicobacter pylori* eradication failure in this study.

## References

[1] Frugis S, Czecko NG, Malafaia O, Parada AA, Poletti PB, Secchi TF (2016). Prevalence of Helicobacter pylori ten years ago compared to the current prevalence in patients undergoing upper endoscopy. ABCD Arq Bras Cir Dig.

[2] Wong F, Rayner-Hartley E, Byrne MF (2014). Extraintestinal manifestations of Helicobacter pylori: A concise review. World J Gastroenterol.

[3] Queiroz DM, Dani R, Silva LD, Santos A, Moreira LS, Rocha GA (2002). Factors associated with treatment failure of Helicobacter pylori infection in a developing country. J Clin Gastroenterol.

[4] Coelho LGV, Marinho JR, Genta R, Ribeiro LT, Passos MDCF, Zaterka S, Assumpção PP (2018). IVth Brazilian Consensus Conference on Helicobacter pylori infection. Arq Gastroenterol.

[5] Shirin H, Gonzalez JO, Hazan S, Gottlieb G, Friedenberg K, Gatof D (2019). The validity of breath collection bags using the novel BreathID® Hp Lab System: a multicenter clinical study in 257 subjects. Ther Adv Gastrointest Endosc.

[6] Nocon M, Kuhlmann A, Leodolter A, Roll S, Vauth Christoph, Willich SN (2009). Efficacy and cost-effectiveness of the 13C-urea breath test as the primary diagnostic investigation for the detection of Helicobacter pylori infection compared to invasive and non-invasive diagnostic tests. GMS Health Technol Assess.

[7] Leal YA, Flores LL, Fuentes-Pananá EM, Cedillo-Rivera R, Torres J (2011). 13C-Urea Breath Test for the Diagnosis of Helicobacter pylori Infection in Children: A Systematic Review and Meta-Analysis. Helicobacter.

[8] Kokkola A, Rautelin H, Puolakkainen P, Sipponen P, Färkkilä M, Haapiainen R (2000). Diagnosis of Helicobacter pylori infection in patients with atrophic gastritis: comparison of histology, 23.C-urea breath test, and serology. Scand J Gastroenterol.

[9] Gisbert JP, Pajares JM (2004). Review article: 13C-urea breath test in the diagnosis of Helicobacter pylori infection a critical review. Aliment Pharmacol Ther.

[10] Shah SC, Iyer PG, Moss SF (2021). AGA Clinical practice update on the management of refractory Helicobacter pylori Infection: expert review. Gastroenterology.

[11] Camargo MC, Piazuelo MB, Mera RM, Fontham ET, Delgado AG, Yepez MC (2007). Effect of smoking on failure of H. pylori therapy and gastric histology in a high gastric cancer risk area of Colombia. Acta Gastroenterol Latinoam.

[12] Namiot DB, Leszczyńska K, Namiot Z, Kurylonek AJ, Kemona A (2008). Smoking and drinking habits are important predictors of Helicobacter pylori eradication. Adv Med Sci.

[13] Akara Y, Endo H, Nakano R, Kawachi K, Hidaka H, Matsunaga T. (2019). Smoking and Drinking Did Not Increase the Failure of Therapeutic Helicobacter pylori Eradication by Vonoprazan, Clarithromycin, and Amoxicillin. Digestion.

[14] Itskoviz D, Boltin D, Leibovitzh H, Tsadok Perets T, Comaneshter D, Cohen A (2017). Smoking increases the likelihood of Helicobacter pylori treatment failure. Dig Liver Dis.

